# Defining the Role of Postoperative Radio-Hormone Therapy in Prostate Cancer

**DOI:** 10.1007/s11912-025-01694-y

**Published:** 2025-06-13

**Authors:** Harshitha Dudipala, Mai Dabbas, Kshitij Pandit, Sarika D. Gurnani, Rana R. McKay

**Affiliations:** 1 Department of Medicine, Moores Cancer Center, University of California San Diego, La Jolla, CA USA; 2 Department of Urology, Moores Cancer Center, University of California San Deigo, La Jolla, CA USA; 3 Department of Medicine, Boston University Medical Center, Boston, MA USA

**Keywords:** Radio-Hormone Therapy, Radical Prostatectomy, ADT, Salvage RT, Adjuvant RT

## Abstract

**Purpose of Review:**

Postoperative radio-hormone therapy plays a significant role in management of prostate cancer after radical prostatectomy (RP), particularly in efforts to reduce biochemical recurrence (BCR), distant metastasis, and improve overall survival. BCR rates can be upwards of 50–70% at 5 years, highlighting the need for optimized risk stratification and consideration of multimodal treatment approaches. The purpose of this review is to highlight evidence-based treatment recommendations, and call attention to the importance of personalized therapeutic strategies after RP.

**Recent Findings:**

Both radiotherapy (RT) and ADT have been shown to optimize survival outcomes and to reduce disease progression in patients with persistent PSA, pathologic lymph-node positive disease, and adverse pathology. Early salvage RT (SRT) is typically a preferred treatment approach as it allows for treatment intensification only when clinically indicated, avoiding unnecessary radiation in men who may never recur. ADT is often added to external beam radiation therapy (EBRT) to enhance treatment efficacy, particularly in patients with high-risk features, though in selected lower-risk scenarios, radiation alone may be sufficient. Short-term ADT is appropriate for low-intermediate risk patients and long-term is appropriate for patients with advanced pathological features or nodal involvement. For certain high-risk pathologic findings, such as positive surgical margins and seminal vesicle invasion (T3b), adjuvant RT (aRT) may be indicated to optimize disease control.

**Summary:**

Overall, radio-hormone therapy plays a significant role in the postoperative setting by reducing the risk of recurrence and disease progression, and improving survival outcomes. There are several well-validated tools that may offer personalized risk assessments to identify which patients may most benefit from adjuvant or salvage therapies. Finally, the optimal use of such therapies continues to be investigated with ongoing trials.

## Introduction

Radical prostatectomy (RP) is a common treatment modality in for localized prostate cancer, however a subset of patients with localized disease are at risk of biochemical recurrence (BCR) after RP. Among high-risk patients, BCR rates can be upwards of 50–70% at 5 years, highlighting the need for optimized risk stratification and consideration of multimodal treatment approaches. Postoperative radiotherapy (RT) and hormone therapy plays a significant role in management, particularly in efforts to reduce BCR, distant metastasis, and improve overall survival (OS). Many studies investigate various adjuvant and salvage strategies, with the aim to determine the most optimal approach. Below we review current data that highlights evidence-based treatment recommendations, and call attention to the importance of personalized therapeutic strategies after RP.

## Risk of Recurrence Post-Operatively

### I. Tools Used to Assess Biochemical Recurrence Risk

Accurate risk stratification after RP to predict risk of BCR is pivotal for tailoring postoperative management and identifying candidates for adjuvant or salvage therapies. A variety of predictive tools have been developed, ranging from traditional clinical models to advanced molecular and imaging biomarkers, each offering unique insights into recurrence risk.

It is important to note that the definition of BCR varies across studies, which impacts the interpretation of results. Two commonly used thresholds are PSA ≥ 0.2 ng/mL and PSA ≥ 0.4 ng/mL on two different measurements after RP [1, 2]. The more common of the two, PSA ≥ 0.2 ng/mL, is used by the American Urological Association, European Association of Urology, the CAPRA-S score, and several major trials (NRT/RTOG 9601, GETUG-AFU 17, SWOG S9921) [3]. The threshold of PSA ≥ 0.4 ng/mL is notably used by the MSKCC Nomogram as well as other major trials (for example, TROG 08.03/ANZUP RAVES) [4]. This variability in definition does limit the direct comparisons that can be made across studies. Moreover, a lower threshold will increase a study’s sensitivity and result in higher reported recurrence rates, which may limit its prognostic accuracy to some degree. A higher threshold on the other hand will result in lower reported recurrence rates, but may improve a study’s prognostic accuracy [5].

#### Post-operative Clinical Tools

Prognostic tools such as the Gleason score, pathological staging, and post-operative prostate specific antigen (PSA) levels form the cornerstone of recurrence risk assessment. Independent clinical variables provide fundamental prognostic information. However, predictive models that integrate these parameters offer a more comprehensive recurrence risk assessment. Several well-validated post-operative models are commonly used in clinical practice. The Han Tables estimated the probability of BCR after RP based on factors such as surgical Gleason score, pathological stage, and PSA, providing recurrence probability estimates for up to 10 years [6].

The Memorial Sloan Kettering Cancer Center (MSKCC) nomograms provide three models tailored for different stages of prostate cancer management: two preoperative models and one postoperative model. The MSKCC Postoperative Nomogram, first developed in 1999, integrates key pathological factors, including surgical Gleason grade, seminal vesical invasion (SVI), extracapsular extension (ECE), lymph node invasion (LNI), and surgical margin status to estimate BCR risk after surgery [7, 8]. For example, the 5-year BCRFS for organ-confined disease, negative margins, and Gleason ≤ 6 was 90–95%; organ-confined disease, positive margins was 70–80%; extracapsular extension, negative margins was 65–75%; extracapsular extension, positive margins was 45–55%; seminal vesicle invasion was 25–35% and lymph node positive disease was 10–20%. The study’s primary endpoint was 5, 7, and 10 year BCR, disease-specific survival (DSS), and metastasis-free survival (MFS). The study demonstrated that 19% experienced recurrence, with a 7-year recurrence-free probability of 73% (95% CI: 68%–76%). DSS at 15 years for those with low-risk features was > 95%, 85–95% in intermediate risk features, 60–80% in high-risk features, and 50–70% in lymph node positive disease. The nomogram exhibited high predictive accuracy, with an AUC of 0.89 in both internal and external validation cohorts, outperforming preoperative models in predicting recurrence risk. This validation confirms the clinical utility of the postoperative nomogram in guiding patient counseling, postoperative surveillance, and decisions regarding adjuvant therapy. Recent studies suggest limited added value of PSA density and prostate volume in improving prediction accuracy and therefore should not heavily influence clinical decisions [7].

To address limitations of the original model, the updated postoperative MSKCC nomogram extends predictions to 10 years post-RP [9]. The 10-year progression-free probability (PFP) for the cohort was 79% (95% CI, 75% to 82%), with a c-index of 0.81 and 0.78 in independent validation cohorts. Updates to the model allow for adjustments based on treatment year and adjuvant RT (aRT) use while enabling dynamic risk recalculations based on a patient’s disease-free interval. Among 4,158 patients in the MSKCC-BCM cohort, those disease-free at 7 years had a 9% risk of recurrence over the next 8 years, while those disease-free at 10 years had only a 3% risk over the next 5 years. A multivariable analysis confirmed preoperative PSA (*P* < 0.002), Gleason grade (*P* < 0.0001), ECE (*P* < 0.0001), SVI (*P* < 0.0001), lymph node involvement (*P* = 0.030), and absence of aRT (*P* = 0.046) as significant predictors of recurrence [9]. The refined nomogram remains a highly accurate postoperative tool, enhancing risk stratification, surveillance planning, and treatment decision-making for patients post-RP.

The Surgical Cancer of the Prostate Risk Assessment (CAPRA-S) nomogram, developed in 2011, is a widely utilized tool for predicting prostate cancer outcomes after RP. The CAPRA-S nomogram refines preoperative risk stratification by incorporating postoperative data, including pathological Gleason score, surgical margin status, ECE, SVI, LNI, and PSA levels to provide a more accurate assessment of recurrence risk [10]. CAPRA-S stratifies patients into three risk categories, with higher scores correlating to increased BCR risk. It has demonstrated strong predictive accuracy, with a c-index of 0.77, indicating reliable discrimination between risk groups [10]. Specifically, the 5-year BCR-free survival (BCR-FS) rates are approximately 85% for low-risk (0–2), 65–75% for intermediate risk (3–5), and 35–40% for high-risk (> 6). Beyond BCR prediction, CAPRA-S is also validated for secondary oncologic outcomes, such as predicting metastasis and cancer-specific mortality (CSM), with a c-index of 0.85 and 0.88 respectively [11]. Cooperberg et al. reported that for each one-point increase in CAPRA-S score, subhazard ratio for CSM was 1.42 (95% CI, 1.27–1.60) [10].

The Eggener nomogram was developed in 2011 to predict 15-year prostate cancer-specific mortality (PCSM) following RP. Using a large cohort of 11,521 patients, the model was externally validated on 12,389 patients and demonstrated high predictive accuracy with a concordance index (c-index) of 0.92 [12]. The study found that overall 15-year PCSM was 7% in the modeling cohort and 4% in the validation cohort, with pathological Gleason score, seminal vesicle invasion (SVI), and the year of surgery emerging as the most significant predictors of mortality. Patients with low-risk features, such as organ-confined disease and Gleason ≤ 6, had negligible PCSM (0.2–1.2%), while those with intermediate-risk features, such as Gleason 3 + 4 or extraprostatic extension, had a PCSM of 4.2–10%. In contrast, high-risk features, including Gleason 8–10, seminal vesicle invasion, and lymph node metastasis, were associated with a significantly increased 15-year PCSM, ranging from 15 to 37%. Notably, among 9,557 patients with Gleason ≤ 6 disease, only three deaths occurred over 15 years, reinforcing the excellent prognosis of low-grade, organ-confined prostate cancer [12].

The Pound progression model, developed at Johns Hopkins University, provides an important framework for predicting metastatic progression following BCR after RP [13]. This study followed 1,997 men, identifying 304 who developed BCR, and tracked their long-term outcomes. Among these men, 103 (34%) progressed to metastatic disease, with a median time from BCR to metastasis of 8 years. Once metastasis occurred, the median survival was approximately 5 years, emphasizing the need for early risk stratification. The model demonstrated that Gleason score, time to BCR, and PSA doubling time (PSADT) were critical predictors of metastatic progression. Gleason score strongly correlated with metastasis risk, with men having Gleason scores < 8 showing a 73% probability of remaining MFS at 5 years post-BCR, compared to only 40% in men with Gleason 8–10 tumors. Additionally, time to BCR played a crucial role, as patients who recurred within ≤ 2 years post-RP had a significantly higher risk of distant failure compared to those with later recurrence. Further risk stratification using PSADT (cutoff of 10 months) helped distinguish high- vs. low-risk groups. Among men with Gleason < 8, those with PSADT > 10 months had a 76% probability of remaining metastasis-free at 5 years, whereas those with PSADT < 10 months had only a 35% chance. Interestingly, in men with Gleason ≥ 8, PSADT was not a statistically significant predictor when time to BCR was accounted for, suggesting that tumor grade alone may drive disease progression in high-risk cases [13]. The Pound progression model serves as an essential algorithm, predicting metastatic risk at 3, 5, and 7 years post-BCR, helping guide postoperative surveillance strategies and decisions on salvage therapy.

#### Molecular and Genomic Tools

Molecular and genomic tools such as the Decipher genomic classifier provide additional layers of risk stratification. Decipher is a 22-gene genomic classifier (GC) that utilizes whole-transcriptome microarray analysis to aid in the prognostication of prostate cancer (PCa) across diverse disease phenotypes [14]. The test employs a random forest algorithm (locked since 2013) to analyze RNA expression from 22 biomarkers associated with androgen receptor signaling, cell proliferation, differentiation, motility, and immune modulation. The Decipher score (ranging from 0 to 1.0) was primarily developed and validated to predict the risk of metastasis. Its applications to other outcomes represent extensions of its original intended use, such as prostate CSM (PCSM), and response to therapy, aiding in treatment decision-making [15]. Risk categories are stratified as follows: low-risk (< 0.45), intermediate risk (0.45–0.60), and high-risk (> 0.60).

The Decipher score significantly stratifies patient outcomes following salvage RT (SRT) [15]. The 12-year cumulative incidence of distant metastasis (DM) was highest in the high-risk group (15.3%; 95% CI, 6.9%–23.7%), followed by the intermediate-risk group (8.7%; 95% CI, 3.7%–13.6%), and lowest in the low-risk group (6.2%; 95% CI, 2.2%–10.1%) (*P* = 0.003). Similarly, PCSM rates were 9.8% (95% CI, 2.9%–16.8%) for high-risk, 2.4% (95% CI, 0.0%–5.0%) for intermediate-risk, and 0.7% (95% CI, 0.0%–2.0%) for low-risk patients (*P* < 0.001) [15]. These findings confirm that Decipher effectively differentiates patients based on recurrence risk, supporting its integration into post-RP treatment planning.

Thus far, Decipher’s predictive utility for treatment response is based on retrospective analyses of prospective trials, and there are ongoing prospective trials specifically designed to evaluate Decipher as a predictive biomarker such as NRG-GU002 and the GUIDANCE study [16, 17]. Retrospective analyses suggest that Decipher scores may help guide the use of ADT in SRT settings, though this remains a hypothesis that requires prospective validation. Analyses of the NRG/RTOG 9601 and SAKK 09/10 trials suggest that patients with high Decipher scores may derive greater benefit from combining ADT with SRT compared to those with low Decipher scores [15, 18]. These findings highlight Decipher’s role in guiding both ADT use and the optimal timing of salvage therapy, ensuring individualized, risk-adapted treatment approaches.

Another widely used genomic tool, the Prolaris test, is a cell cycle progression (CCP) score-based assay that quantifies tumor aggressiveness based on the expression of genes involved in cell proliferation [19]. The CCP score has been validated in post-RP patients to predict BCR risk, distant metastasis, and PCSM. Cooperberg et al. found that each unit increase in CCP score was associated with a doubling of the risk of recurrence (HR: ~ 2.0, *p* < 0.001) [20]. When combined with the CAPRA-S score, the predictive accuracy improved significantly, with a C-index increasing from 0.73 to 0.77 (*P* < 0.001), demonstrating that CCP adds significant prognostic value beyond standard clinicopathologic factors.

Emerging biomarkers, such as tissue-based microRNA signatures, are also being investigated for their predictive potential. These biomarkers could refine active surveillance criteria, particularly in intermediate-risk patients, by offering more personalized risk assessments [21].

#### Advanced Imaging Techniques

PSMA PET imaging has emerged as a pivotal tool in the preoperative assessment of prostate cancer, offering superior accuracy in detecting both local and distant disease. A phase 3 diagnostic trial evaluating 764 men with intermediate to high-risk prostate cancer found that among the 277 patients who underwent RP with lymph node dissection, the sensitivity and specificity of PSMA PET for detecting pelvic nodal metastases were 40% and 95%, respectively, thus demonstrating its high specificity for nodal staging pre-operatively [22]. Similarly, another prospective study evaluating PSMA PET vs mpMRI found that PSMA-PET had higher sensitivity (86% vs. 57%) for detecting EPE along the neurovascular bundle compared to mpMRI [23]. These findings suggest that PSMA-PET enhances preoperative planning and may improve functional outcomes by reducing unnecessary nerve resection in high-risk prostate cancer cases.

In the postoperative setting as well, PSMA PET has demonstrated significant utility in guiding SRT decisions. A phase 3 randomized trial evaluating PSMA PET in SRT planning in 193 patients with BCR post-prostatectomy found that PSMA-PET led to major treatment changes in 33% of patients, including a 23% higher rate of management modifications and a 17.6% higher rate of treatment escalation thus demonstrating its significant role in refining SRT planning after prostatectomy [24].

### II. Risk of Recurrence

BCR occurs in 20–40% of patients postoperatively, depending on tumor characteristics and treatment approach [25]. High-risk features such as Gleason score ≥ 8, postoperative short PSA doubling time (< 6 months), and T3b stage further elevate the likelihood of recurrence [26, 27]. Patients with persistent PSA, pathologic lymph node-positive (LN +) disease, or adverse pathologic features such as T3b stage or positive surgical margins face significantly elevated risks of recurrence following RP [28]. Persistent PSA, defined as a PSA level ≥ 0.1 ng/mL within 4–8 weeks post-surgery, occurs in 8–26% of patients and is strongly associated with poor long-term outcomes, including increased risks of BCR, metastases, and CSM [26, 29].

Pathologic LN + disease compounds recurrence risk further, especially in patients with three or more positive nodes. Studies show that patients with three or more LN + have significantly worse recurrence-free survival (RFS) at 3 and 5 years (38.6% and 32.1%, respectively) compared to those with fewer positive nodes (60.8% and 48.9%) [30]. Similarly, patients with T3b disease or positive surgical margins face elevated recurrence risks, with bilateral SVI associated with particularly poor biochemical recurrence-free survival (BCRFS) and cancer-specific survival (CSS) [31]. The 5-year BCRFS was markedly lower for bilateral SVI (16.64%) compared to unilateral SVI (31.03%), indicating nearly double the risk of BCR in patients with more extensive disease. Similarly, the 5-year CPFS was 78.09% for bilateral SVI versus 87.46% for unilateral SVI, showing a higher rate of disease progression in patients with bilateral involvement. CSS at 5 years remained high in both groups but was still lower in bilateral SVI cases (94.34%) compared to unilateral SVI (98.50%), underscoring the increased risk of cancer-related mortality in patients with bilateral invasion.

## Select Scenarios

### Persistent PSA

Multivariable Cox Regression Analysis (MVA) [MOU1] has identified persistent PSA as a significant predictor of BCR (HR: 5.47, *p* < 0.001), among other adverse outcomes in prostate cancer. High rates of persistent PSA are linked to adverse pathological features including positive surgical margins, LNI, or pT3b–4 stage. Key predictors include higher preoperative PSA levels (OR: 1.04, *p* = 0.02), biopsy Gleason scores of 8–10 (OR: 1.81, *p* = 0.01), and advanced pathologic stage ≥ T3b (OR: 1.78, *p* = 0.02) [26].

Persistent PSA is associated with significantly worse 10-year cancer progression-free survival (CPFS: 63.8% vs. 93.5%), CSS (78.5% vs. 98.3%), and overall survival (OS: 54% vs. 83.2%) compared to those with undetectable PSA (all *p* < 0.001) [32]. Management strategies for persistent PSA include SRT with or without ADT, ADT, or observation. SRT, particularly when combined with ADT, demonstrates superior outcomes compared to ADT monotherapy, offering better cancer control and reducing the progression to castration-resistant prostate cancer (CRPC) [32, 33]. Propensity score-matched analysis demonstrated superior castration-resistant prostate cancer-free survival rates (CRPC-FS) for SRT compared to ADT, with 10- and 15-year rates of 94.1% versus 75.9% and 94.1% versus 58.5% (*p* = 0.017). In contrast, ADT monotherapy is associated with higher risks of worse CSS (HR: 3.9, *p* = 0.005) and OS (HR: 4.7, *p* < 0.0001), underscoring the importance of integrating RT in high-risk cases [33].

### Pathologic LN Positive (LN +) Disease

Recent advancements in the management of prostate cancer with pathologic LNI highlight the critical role of postoperative radio-hormonal therapy. Shiota et al. emphasize the prognostic importance of parameters like International Society of Urologic Pathology (ISUP) grade group, PSA levels at diagnosis, pathologic T-stage, and surgical margin status in predicting RFS and MFS [30]. Their model stratified patients into low- and high-risk groups, revealing significant survival differences, with low-risk patients demonstrating more favorable outcomes even with three or more positive lymph nodes [30].

In patients with prostate cancer exhibiting pathologic lymph node involvement (pN1) following RP, the combination of postoperative RT and ADT has been associated with improved CSS [34]. Retrospective studies indicate that approximately one-third of these patients experience clinical recurrence, often manifesting first in the prostate bed and pelvic lymph nodes. Administering postoperative RT alongside ADT has been shown to enhance survival outcomes in this cohort [34].

Messing et al. demonstrated that immediate [MOU2] ADT significantly improves survival in patients with pN1 disease [35]. This clinical trial included patients with pN1 disease who had undergone RP and pelvic lymphadenectomy, and compared outcomes for those who received up-front ADT as opposed to delayed ADT. It is important to note that this landmark study used an observation approach that differs from modern surveillance protocols, as delayed ADT was only initiated upon detection of distant metastases or symptomatic recurrences rather than at biochemical recurrence. Though not standard practice, immediate ADT significantly improved OS (hazard ratio (HR) 1.84, *p* = 0.04), prostate cancer-specific survival (HR 4.09, *p* = 0.0004), and progression-free survival (HR 3.42, *p* < 0.0001) [35]. Furthermore, Washington et al. noted that while more extensive lymph node dissection was not associated with a greater risk of BCR the degree of nodal positivity was [36]. They also found that neither more extensive lymph node dissection at RP nor adjuvant treatment after salvage therapy were significantly associated with improved OS or prostate cancer-specific survival [36]. These findings collectively highlight the complexity of treatment strategies, and suggest that personalized treatment approaches integrating radio-hormonal therapy, risk stratification, and advanced imaging may optimize outcomes for patients with pN1 prostate cancer.

### T3b/Positive Surgical Margins

In patients with prostate cancer characterized by seminal vesicle invasion (pT3b) or positive surgical margins (R1) following radical prostatectomy, adjuvant therapy has been found to be beneficial. As such, the integration and timing of postoperative RT and ADT has been extensively studied to enhance oncological outcomes. The optimal use of RT and ADT has been the subject of extensive research, emphasizing the importance of tailoring treatment strategies to maximize outcomes while minimizing toxicity.

Patients with SVI, particularly bilateral SVI, are at heightened risk for BCR, cancer progression (CP), and reduced CSS [31]. The high rates of extracapsular extension, lymph node metastasis, and positive resection margins in this cohort underscore the necessity of tailored postoperative therapies. Suh et. al found that patients with bilateral SVI more frequently received adjuvant ADT (19%) compared to those with unilateral SVI (11.5%, *p* = 0.005) [31]. Similarly, bilateral SVI patients more frequently required salvage ADT (65.1% vs. 54.7%; *p* = 0.004). Bilateral SVI was independently associated with worse outcomes in covariate-adjusted Cox regression analyses: BCR-free survival: HR, 1.197; *p* = 0.049, CP-free survival: *p* = 0.022, and CSS: *p* = 0.038 [31].

The addition of ADT to RT has demonstrated enhanced efficacy in improving survival outcomes. Trials such as SPPORT and SALV-ENZA showed that combining SRT with short-term ADT (4–6 months) significantly improved BCR-free survival [37, 38]. In the RTOG 9601 trial, 24 months of bicalutamide with SRT resulted in a significant improvement in OS for men with adverse features, including T3b disease and positive margins (HR, 0.77; *P* = 0.04) [39]. These findings highlight the importance of hormonal therapy as an adjunct to SRT, with longer ADT courses showing greater benefits in high-risk populations.

The timing of SRT relative to PSA levels is a critical determinant of outcomes. Evidence suggests that initiating SRT at lower PSA levels (< 0.2 ng/mL) is associated with better BCRFS [40]. Furthermore, intensified RT doses (> 70 Gy) may provide incremental benefits for patients who undergo delayed SRT at higher PSA levels [41].

### Adjuvant Versus Salvage External Beam Radiation Therapy (EBRT)

The role of postoperative EBRT in prostate cancer remains an area of ongoing clinical consideration, particularly regarding the timing of RT after RP. The two primary approaches include aRT, administered immediately after surgery in patients with adverse pathology, and SRT, initiated at the time of BCR.

aRT is delivered after RP primarily based on adverse pathological features (such as positive surgical margins, extracapsular extension, seminal vesicle invasion, or lymph node involvement), typically when PSA is undetectable but can sometimes be administered with detectable PSA levels [42]. Several early randomized trials showed that aRT for patients with these features improves oncological outcomes. The EORTC 22911 trial compared aRT to delayed salvage RT in 1,005 men with pT3 disease or positive margins, and showed that while aRT significantly improved biochemical progression-free survival (bPFS), it was not associated with improved OS compared to the delayed salvage RT group [43]. These findings were validated by the ARO 96–02/AUO AP 09/95 trial which evaluated aRT vs observation in 307 patients with pT3 disease and demonstrated that bPFS after 5 years was 72% in the aRT group compared to 54% in the observation group [44]. Subsequently, the SWOG 8794 trial, which randomized 425 patients with high-risk pathology at prostatectomy to aRT vs observation showed the patients in the aRT arm were associated with a significantly higher metastasis-free and OS [42]. Despite promising results, aRT is associated with potential overtreatment, particularly in patients who may not experience disease recurrence, prompting studies to evaluate early SRT.

SRT allows for treatment stratification, avoiding unnecessary radiation in men who may never recur. A retrospective study by Hervás et al. evaluating 702 post-prostatectomy patients who received either aRT (223) or SRT (479) found that aRT was associated with a better biochemical relapse-free survival at 2 and 5 years with compared to SRT. However, there was no significant difference in OS between the two modalities [45]. Subsequently, there have been three randomized trials comparing aRT and SRT, providing important implications for the management of recurrent prostate cancer. The RAVES study, which was a Phase 3 trial comparing 166 patients receiving aRT vs 167 patients receiving SRT found that there was no significant difference in the freedom from 5-year biochemical progression between both groups. Additionally, the genitourinary adverse effects were found to be lower in the SRT (54%) cohort compared to the aRT cohort (70%) [46]. Around the same time, GETUG-AFU 17 which was a multicenter trial evaluating 424 patients found that there was no significant difference in the EFS between the aRT group (92%) and the SRT group (90%). They also found that the incidence of late genitourinary adverse effects and erectile dysfunction was higher in the aRT group compared to the SRT group [47].

Subsequently, the RADICALS-RT trial is the largest randomized control trial (1,396 patients) to date comparing aRT vs. SRT. This trial randomized patients with at least one risk factor for recurrence after RP (including but not limited to pT3/4 disease, Gleason score 7–10, positive margins, pre-operative PSA ≥ 10 ng/ml, or pN1) to either aRT or to observation with SRT for PSA failure [48]. It demonstrated no significant differences in 5-year BCR-free survival (BCRFS) between aRT and SRT, with BCRFS rates of 85% and 88%, respectively. However, aRT was associated with higher rates of urinary incontinence and bowel morbidity, supporting the current standard of observation with SRT triggered by rising PSA levels [48].

Finally, a systematic review and meta-analysis pooling the findings from these three trials showed that aRT does not improve EFS in localized or locally advanced prostate cancer, suggesting that early SRT would be the preferred treatment approach as it prevents radiation related adverse effects in numerous patients who do not relapse [49]. Additional studies have found the timing of SRT to affect treatment outcomes, as patients with very early SRT (PSA < 0.2 ng/mL) had higher 5-year hormone therapy-free survival rates (98% for low-intermediate risk vs. 88% for high-risk) than those receiving delayed SRT (PSA > 0.5 ng/mL), where survival rates dropped to 83% and 54%, respectively [18]. Thus, the paradigm of postoperative RT is shifting toward a salvage-first approach, driven by randomized trials and meta-analyses.

### Role of ADT in Conjunction with EBRT

ADT is often added to EBRT to enhance treatment efficacy, particularly in patients with high-risk features as demonstrated in several randomized control trials. The SPPORT trial recruited patients post-RP with a persistently detectable or rising PSA and compared men who received EBRT alone to those who received EBRT with short-term ADT (4–6 months). Those who received short-term ADT had significantly lower rates of biochemical progression (adjusted HR 0.63, *p* = 0.00015) [37]. The GETUG-AFU 16 trial similarly compared patients who received EBRT to those who received EBRT in addition to short-term androgen suppressive therapy. Results showed that patients who received short-term androgen suppression had significantly lower rates of biochemical progression (HR 0.50, *p* < 0.0001) [50]. For low-intermediate risk, short-term ADT (4–6 months) is typically sufficient. Long-term ADT (18–36 months) may be considered in patients with advanced pathological features or nodal involvement [39].

For example, The RTOG 9601 trial evaluated the efficacy of adding long-term ADT, bicalutamide, to SRT in men with recurrent prostate cancer after RP. The trial included men with a positive surgical margin or pathologic T3 disease and pre-SRT PSA levels of 0.2 to 4.0 ng/mL. The study highlighted that 24 months of bicalutamide improved both MFS and OS in patients receiving adjuvant EBRT [39]. RADICALS-HD was an international RCT that compared outcomes of no ADT, short-course ADT (6 months), and long-course ADT (24 months) in patients who were indicated for postoperative RT after radical prostatectomy. The trial demonstrated that long-course ADT significantly improved MFS compared to short-course ADT, suggesting that patients who can tolerate the additional adverse effects should consider long-course ADT [51]. However, the improvement in MFS did not translate to OS benefits during a median follow-up of nine years, emphasizing the need to balance the prolonged adverse effects of ADT with its benefits.

FORMULA-509 investigated the utility of adding abiraterone/prednisone and apalutamide (AAP/Apa) to SRT and short-term ADT in patients with unfavorable features [52]. The study strongly suggested that the addition of AAP/Apa improves PFS and MFS though did not reach statistical significance. The 3-year PFS was 74.9% for AAP/Apa and 68.5% for controls (HR 0.71, *p* = 0.06). The 3-year MFS was 90.6% for AAP/Apa and 87.2% for controls (HR 0.57, *p* = 0.05). A pre-planned analysis did show statistically significant benefit for AAP/Apa in patients with PSA > 0.5 for both PFS and MFS [52].

Ultimately the duration and intensity of both EBRT and ADT depend on patient-specific factors. Contemporary protocols recommend dose-escalated EBRT (≥ 66 Gy) for salvage settings and 70–72 Gy for adjuvant therapy, depending on the target volume and anatomical considerations [37, 39]. While short-term ADT suffices for most cases, prolonged ADT may be necessary for patients with higher Gleason scores or nodal involvement [48]. Overall, continuous ADT is the standard approach when combined with salvage RT.

### Role of ADT Monotherapy

The role of ADT monotherapy after radical prostatectomy has been a topic of discussion, especially in those with BCR or high-risk features. The American Urological Association (AUA) and the American Society for Radiation Oncology (ASTRO) guidelines suggest that adjuvant ADT may be offered to patients with high-risk features after RP, especially those with LN + disease, to improve PFS and OS [53]. A study by Siddiqui et al. showed that adjuvant ADT in patients with pathological T3b prostate cancer improved 10-year bPFS (60% vs. 16%), local RFS (87% vs. 76%), systemic PFS (91% vs. 78%), and CSS (94% vs. 87%) compared to those who did not receive adjuvant ADT [54].

The SWOG S9921 study investigated the role of adjuvant ADT with or without the addition of mitoxantrone plus prednisone (MP) in patients with high-risk prostate cancer after RP. After a median follow-up of 11.2 years, the 10-year OS estimates were 87% for the ADT arm and 86% for the ADT + MP arm (HR: 1.06; 95% CI: 0.79–1.43), therefore demonstrating that the addition of MP to adjuvant ADT did not improve OS [55].

In the salvage setting, it is important to consider the upper limit of PSA levels where there are diminishing returns on RT for patients with high-risk prostate cancer. The RAVES and RADICALS-RT trials have provided valuable insights into PSA thresholds and outcomes for SRT, though they primarily focused on early salvage intervention rather than defining an upper PSA threshold where treatment becomes futile. RADICALS-RT (with 1,396 patients) showed progressively declining biochemical control rates as PSA increased, with freedom from biochemical progression rates of 88% for PSA ≤ 0.2 ng/mL, decreasing to 63% for PSA > 1.0 ng/mL [48]. Similarly, RAVES (333 patients) demonstrated 5-year freedom from biochemical failure rates of 86% for PSA ≤ 0.2 ng/mL, declining to 69% for PSA > 0.5 ng/mL [46]. However, for defining the upper PSA threshold where SRT is no longer beneficial, we must look to other studies such as RTOG 9601 and the Stephenson multi-institutional analysis. RTOG 9601 included patients with PSA up to 4.0 ng/mL and showed significantly worse outcomes in patients with PSA > 4.0 ng/mL, even with the addition of hormone therapy [39]. The Stephenson analysis demonstrated that biochemical control rates dropped below 20% when PSA exceeded 4.0 ng/mL at the time of SRT [56]_._ These findings, combined with data from GETUG-AFU 16 (which included patients up to PSA 2.0 ng/mL), suggest that SRT alone provides minimal benefit when PSA exceeds 4.0–5.0 ng/mL, at which point patients should be considered for systemic therapy or clinical trials due to the high likelihood of occult metastatic disease [50].

### Role of Chemohormonal Therapy

The role of chemotherapy post-operatively has primarily been explored in high-risk patients. The Veteran Affairs Cooperative Studies Program Study #553 investigated the use of adjuvant chemotherapy for patients with high risk of relapse after prostatectomy. The study found no statistically significant difference in PFS between the docetaxel + prednisone group versus observation group (55.5 months vs. 42.2 months, respectively; HR 0.80; 95% CI 0.58–1.11; *p* = 0.18) [57]. Although, prespecified subgroup analyses showed benefit in PFS for those with tumor stage ≥ T3b and Gleason score ≤ 7.

The lack of benefit in the general high-risk population limits the use of routine chemotherapy. Furthermore, the AUA, ASTRO, and SUO recommend against routine use of salvage chemotherapy with docetaxel in patients undergoing SRT and ADT, due to lack of benefit and increased toxicity [58].

### Key Ongoing Clinical Trials

There are a few key ongoing trials investigating the role of post-operative RT and hormone therapy in prostate cancer. The BALANCE (NCT03371719) trial is investigating whether SRT with apalutamide will improve bPFS compared to SRT alone in patients post-prostatectomy with BCR [59]. Similarly, the PRESTO clinical trial randomized patients with high-risk biochemically relapsed castration-sensitive prostate cancer (BRPC) after RP into three arms: ADT alone, ADT plus apalutamide, and ADT plus apalutamide and abiraterone acetate plus prednisone (AAP). The trial supported the use of intensified androgen receptor blockage, with median PSA-PFS of 24.9 months for ADT plus apalutamide vs. 20.3 months for ADT alone (HR, 0.52; 95% CI, 0.35 to 0.77; *P* = 0.00047), and 26.0 months for ADT plus apalutamide and AAP vs. 20.0 months for ADT alone (HR, 0.48; 95% CI, 0.32 to 0.71; *P* = 0.00008) [60]. Furthermore, INNOVATE (NRG-GU008) is a randomized phase III trial evaluating SRT combined with ADT with or without the addition of abiraterone and apalutamide in patients with node-positive prostate cancer after radical prostatectomy [61]. Overall, these trials are crucial in improving patient outcomes by helping determine the optimal use of RT and hormone therapy post-operatively in prostate cancer.

## Conclusion

Radio-hormone therapy plays a critical role in management of prostate cancer in the postoperative setting. There are several well-validated tools, including those that incorporate new imaging modalities and biomarkers, that may offer personalized risk assessments to identify which patients may most benefit from adjuvant or salvage therapies, particularly in patients with high-risk disease. Both RT and ADT have been shown to optimize survival outcomes and to reduce disease progression in patients with persistent PSA, pathologic lymph-node positive disease, and adverse pathology (stage T3b or with positive surgical margins). In high-risk patients, SRT is associated with lower rates of mortality than aRT, and longer ADT courses show greater benefits in these high-risk populations. EBRT improves progression-free survival and delays metastases, though salvage EBRT limits unnecessary treatment in patients who do not experience recurrence compared to adjuvant EBRT. ADT may be added to EBRT to enhance treatment efficacy, especially for patients with high-risk features. Short-term ADT is appropriate for low-intermediate risk patients and long-term is appropriate for patients with advanced pathological features or nodal involvement. In the salvage setting, both literature to date and current guidelines recommend a combination of ADT and SRT for improved survival outcomes. Overall, postop radio-hormone therapy plays a significant role in the management of prostate cancer, and ongoing trials are imperative in determining the optimal use of such therapies.

## Key References

- Dal Pra A, Ghadjar P, Hayoz S, et al. Validation of the Decipher genomic classifier in patients receiving salvage radiotherapy without hormone therapy after radical prostatectomy - an ancillary study of the SAKK 09/10 randomized clinical trial. Ann Oncol. 2022;33(9):950–958. doi:10.1016/j.annonc.2022.05.007
  - ○ This is the first contemporary randomized controlled trial that has validated the prognostic utility of the Decipher genomic classifier in patients treated with early SRT without concurrent hormone therapy or pelvic nodal RT.
- Bahler CD, Tachibana I, Tann M, et al. Comparing Magnetic Resonance Imaging and Prostate-Specific Membrane Antigen-Positron Emission Tomography for Prediction of Extraprostatic Extension of Prostate Cancer and Surgical Guidance: A Prospective Nonrandomized Clinical Trial. *J Urol*. 2024;212(2):290–298. doi:10.1097/JU.0000000000004032
  - ○ The study highlights the complementary roles of PSMA PET/CT and mpMRI in the context of surgical planning and staging.
- Komori T, Matsumoto K, Kosaka T, et al. Long-Term Prognosis and Treatment Strategy of Persistent PSA After Radical Prostatectomy. *Ann Surg Oncol*. 2023;30(11):6936-6942. doi:10.1245/s10434-023–13780-1
  - ○ This study underscores that persistent PSA after RP is a poor prognostic marker, and optimal treatment should include SRT.
- Milonas D, Laenen A, Venclovas Z, Jarusevicius L, Devos G, Joniau S. Benefits of early salvage therapy on oncological outcomes in high-risk prostate cancer with persistent PSA after radical prostatectomy. *Clin Transl Oncol*. 2022;24(2):371–378. doi:10.1007/s12094-021–02700-y
  - ○ This study highlights that early SRT ± ADT resulted in significantly improved CSS and OS in men with persistent PSA comparing to early ADT monotherapy.
- Ballas LK, Reddy CA, Han HR, et al. Patterns of Recurrence Following Radiation and ADT for Pathologic Lymph Node-Positive Prostate Cancer: A Multi-institutional Study. *Pract Radiat Oncol*. Published online December 27, 2024. doi:10.1016/j.prro.2024.12.006
  - ○ This study underlines the importance of aggressive local and regional treatment strategies in managing pathologic lymph node-positive disease.
- Parker CC, Petersen PM, Cook AD, et al. Timing of radiotherapy (RT) after radical prostatectomy (RP): long-term outcomes in the RADICALS-RT trial (NCT00541047). *Ann Oncol*. 2024;35(7):656–666. doi:10.1016/j.annonc.2024.03.010
  - ○ This large, randomized trial with 1396 participants from the UK, Denmark, Canada, and Ireland found that aRT after RP does not meaningfully improve disease control, and therefore instead an observational policy with SRT should be employed.
- Parker CC, Kynaston H, Cook AD, et al. Duration of androgen deprivation therapy with postoperative radiotherapy for prostate cancer: a comparison of long-course versus short-course androgen deprivation therapy in the RADICALS-HD randomised trial. Lancet. 2024;403(10442):2416-2425. doi:10.1016/S0140-6736(24)00549-X
  - ○ This study provides robust evidence that long-course ADT significantly improved MFS compared to short-course ADT, suggesting that patients who can tolerate the additional adverse effects should consider long-course ADT.

## Data Availability

No datasets were generated or analysed during the current study.
